# Topography of the insular cortex in heart rate control: high-precision mapping reveals critical role of the middle short gyrus

**DOI:** 10.3389/fnins.2025.1665378

**Published:** 2025-09-17

**Authors:** Xiaolin Tian, Jing Wang, Xiongfei Wang, Xue Yang, Tianfu Li, Jiahui Deng, Chongyang Tang, Mengyang Wang, Guoming Luan

**Affiliations:** ^1^Department of Neurology, Sanbo Brain Hospital, Capital Medical University, Beijing, China; ^2^Department of Neurosurgery, Sanbo Brain Hospital, Capital Medical University, Beijing, China; ^3^Chinese Institute for Brain Research, Beijing, China; ^4^Beijing Key Laboratory of Epilepsy, Beijing, China; ^5^Epilepsy Institution, Beijing Institute of Brain Disorders, Beijing, China

**Keywords:** neurostimulation, insula, heart rate variability, intracranial electrical stimulation, SUDEP

## Abstract

**Aims:**

Although accumulating evidence has demonstrated the involvement of the insular cortex in autonomic regulation, the precise functional topography of insular subdivisions mediating cardiovascular control remains controversial. This study aimed to investigate the role of the insula, with high anatomic precision, in regulating heart rate (HR).

**Methods:**

Overall, 487 electrical stimulations (E-stim) in patients with refractory epilepsy were used to investigate the incidence of evoked cardiac response after insula E-stim. Correlations between the parameters of E-stim, insula subdivision, and the HR shift profile were analyzed.

**Results:**

Briefly, 40.8% of insula stimulations evoked a cardiac response. The left insula was more likely to induce cardiac responses. Compared to the electrode contacts in the declining HR group, those in the elevated HR group were predominantly distributed in the posterior insula. Notably, the middle short gyri can rapidly elicit a significant decrease in HR.

**Conclusion:**

Our findings suggest that the middle short gyrus of the insula may serve as the primary cortical region mediating HR reduction. This provides new insights for the prevention and treatment of arrhythmias and sudden unexpected death in epilepsy (SUDEP).

## Graphical abstract

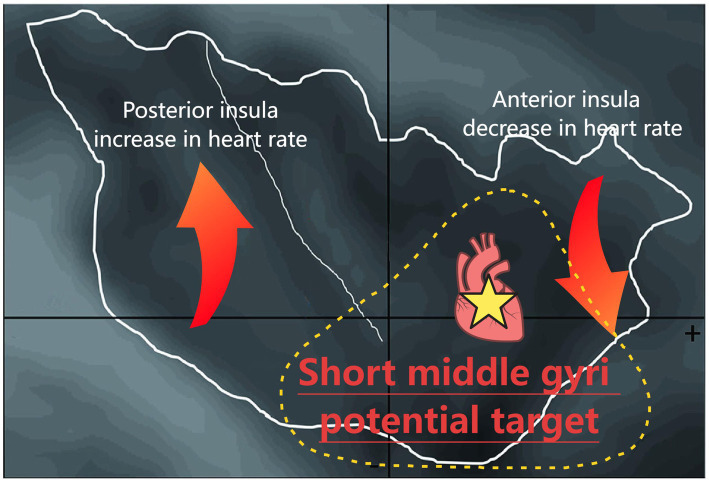

## 1 Introduction

The autonomic nervous system (ANS) comprises the sympathetic and parasympathetic nervous systems, which regulate visceromotor, neuroendocrine, pain perception, behavioral responses and organ activities (Wehrwein et al., 2016). Cardiovascular homeostasis maintained by the ANS is essential for survival (Mohanta et al., 2023; Silvani et al., 2016), and is mainly characterized by heart rate (HR) regulation, blood pressure modulation, and blood flow redistribution. The cortical areas that are involved in the central control of the ANS include the insular cortex, amygdala, hypothalamus, and periaqueductal gray matter, and together they form the neural network termed the central autonomic network (CAN) (Benarroch, 1993; Ruiz Vargas et al., 2016; Samuels, 2007). The role of the cerebral cortex within the CAN in regulating cardiovascular function requires further clarification.

Recent studies have progressively focused on the insula in the CAN in modulating cardiac activities (Mazzola et al., 2023; Oppenheimer and Cechetto, 2016). However, the respective contributions of insular lateralization and cytoarchitectonic subdivisions to specific cardiac outcomes remain controversial. Evidence suggests right insular damage is positively correlated with sympathetic dysfunction (Olga et al., 2022). Supporting this finding, de Morree et al. (2016) reported that right insular lobectomy significantly increased parasympathetic activity and induced bradyarrhythmia, whereas no changes were observed in the left insular resection. In contrast, Alice et al. (2023) found that atrophy in the left middle-to-posterior insula is associated with impaired parasympathetic activity and cardiac arrhythmias, while degeneration in the left ventral anterior insula correlates with reduced sympathetic tone. Low-frequency ultrasonic stimulation of the anterior insula in healthy individuals was found to alter heart rate variability (HRV), whereas stimulation of the posterior insula elicited no such effect (Legon et al., 2024). However, an electrical stimulation (E-Stim) study reported that both anterior and posterior insular regions participate in autonomic regulation, with anterior stimulation preferentially influencing parasympathetic tone and posterior stimulation affecting sympathetic function (Chouchou et al., 2019). Notably, clinical follow-up studies have identified two cases of sudden unexpected death in epilepsy (SUDEP) in patients with left insular damage. These individuals exhibited progressive alterations in HRV prior to death, suggesting that insular lobe lesions may constitute additional risk factors for SUDEP (Lacuey et al., 2016; Matusik et al., 2023).

Beyond its role as a critical upstream ANS region that requires further study, the insula elicits a spectrum of downstream cardiac effects (Nagai et al., 2010). E-stim studies report direct HR changes and impaired cardiac output (Chouchou et al., 2019; Sanchez-Larsen et al., 2021), while clinical investigations have primarily focused on its role in modulating HR variability (Lacuey et al., 2016; Danilin et al., 2022). In animal models, insular E-Stim induced seizures in 92.3% of rats, accompanied by typical autonomic changes—notably a decrease in HR. Among the rats that exhibited significant bradycardia, post-seizure mortality within 2 weeks reached 25% (Ming et al., 2020). Complementing these findings, non-invasive neuroimaging studies have extensively characterized the insula’s involvement in cardiac interoceptive accuracy and subjective cardiopulmonary sensation (Haruki and Ogawa, 2021; Hassanpour et al., 2018; Chong et al., 2017). Despite these advances, the precise functional organization of insular subregions underlying specific cardiac outcomes remains incompletely resolved.

The association between insular cortex function and HR dynamics has not been systematically investigated in large-scale studies. It remains unclear whether distinct insular subregions can elicit differential cardiac contractility or rhythm responses and how these responses are modulated by with E-Stim parameters. Clarifying these relationships may have important implications for preventing arrhythmias, sudden cardiac death, and enhancing the precision of epileptic focus localization prior to surgery. Direct cortical stimulation via intracranial electrodes offers superior temporal and spatial resolution over non-invasive methods like functional magnetic resonance imaging, thereby providing a unique capacity for causal validation of precise anatomical-functional relationships. The present study aimed to investigate the relationship between insular subregions and cardiac responses to direct insular E-stim, and to explore the roles of E-stim parameters and lateralization.

## 2 Methods

### 2.1 Study design and patient selection

Patients with a preliminary diagnosis of refractory epilepsy admitted to Sanbo Brain Hospital of Capital Medical University from 2021 to 2023 were recruited. The inclusion criteria were: (1) implantation of a stereotactic electrode into the insula; (2) intact ability to express symptoms accurately; and (3) provision of written informed consent. The exclusion criteria were: (1) history of cardiovascular disease such as heart failure or arrhythmia; (2) use of cardiovascular drugs; and (3) prior brain surgery.

To avoid confounding effects of pathological tachycardia that could be caused by abnormal discharges, seizures, or auras during E-Stim, we excluded patients with neuroimaging evidence of insular lesions. Furthermore, we excluded any patient who experienced the followings during insula E-stim: (1) a seizure or seizure aura; (2) persistent spike-slow complex wave discharges observed on Stereoelectroencephalography (SEEG), or (3) any subjective symptoms reported by the participant including somatosensory (paresthesia, thermal, or pain), visceral (constrictive sensation in abdomen or throat, nausea, facial blush, etc.), auditory, gustatory, and olfactory sensations, as well as emotional or memory-related experiences. This study was approved by the local ethics committee and performed in accordance with the Declaration of Helsinki. All patients provided written informed consent.

### 2.2 SEEG electrode implantation and insula localization

All enrolled patients underwent continuous video SEEG monitoring using a 128-lead Nicolite video electroencephalogram monitoring device (128 channels, sampling rate: 512 Hz; Thermo Nicolet Corporation). Electrode implantation in the suspected epileptogenic zone was designed for each patient based on the patient’s clinical history, seizure semiology, MRI, positron emission tomography (PET), magnetoencephalography (MEG), and neuropsychological assessment. The SEEG implantation and monitoring process was performed by an experienced multidisciplinary team including neurosurgeons, neurologists, neurophysiology technicians, neuroradiologists, and anesthesiologists. Stereotactic electrodes (Beijing Huake Henson Medical Technology Co., Ltd.) were used, with 8, 10, 12, or 16 contacts per electrode, according to the clinical situation, a contact diameter of 0.8 mm, and an inter-contact interval of 3.5 mm (Yujiao et al., 2023).

The precise anatomical location of each contact was identified through multimodal image reconstruction and modeling of preimplantation MRI with post-implantation CT under MATLAB software (Stolk et al., 2018). Then, the electrode cluster within the MRI was automatically detected and visually inspected, and its trajectory was used to calculate the spatial coordinates of the electrodes. All E-stim sites were spatially localized using the Talairach coordinate system. The AC-PC distances of all patients were normalized. The stereotactic coordinates of each stimulation site were those of the midpoint between the two adjacent contacts used for bipolar stimulation. In order to expand the sample size and improve statistical power, the sites located in the right hemisphere mirrored those in the left hemisphere.

### 2.3 Electrocardiogram data recording and processing

Electrocardiograms (ECG) were recorded continuously for 24 h during the hospital admission period, simultaneously with SEEG recording. All data were stored for offline analysis by the Nicvue 5.30 analysis system using the bipolar lead contact method. The ECG data was acquired using the bipolar limb lead configuration with electrodes placed on the patients’ bilateral deltoid muscles, using the same amplifier and sampling rate as the SEEG. The ECG signals was preprocessed in MATLAB to remove artifacts, correct the baseline, and filter noise, and then real-time HR detection is performed using the highly modular ECGdeli toolbox (Pilia et al., 2021). For HRV analysis, we selected a stable 10-min segment of ECG recording obtained during a morning resting state immediately after awakening. Baseline HRV was assessed by calculating the root mean square of successive RR interval differences (RMSSD) and the standard deviation of normal-to-normal intervals (SDNN) from this segment.

### 2.4 Cortical functional E-stim process

To clarify the diagnosis and localize the epileptogenic foci, as well as to guide surgical procedures, routine cortical E-Stim mapping was performed when patients had ≥3 habitual seizures on SEEG and after reinstating their baseline anti-seizure medications (ASM) for up to 24 h. The Natus stimulator (Nicolet Cortical Stimulator, WI, USA) was used. Stimulations were applied at 50 Hz with biphasic square waves, a train duration of 5 s, intervals of 5–10 s, and an intensity of between 0.5 and 6 mA. Stimulus intensity started at 0.5 mA and increased in steps of 0.5 mA until any clinical response or until a maximum stimulus intensity of 6 mA was reached. The stimulation threshold was defined as the minimum E-stim intensity required to evoke a clinical response. No stimulation was performed above the threshold. During the stimulation, patients were asked to sit calmly on the bed and perform a verbal count to detect speech impairment. Subjective reports and clinical observations were collected immediately after each stimulation. The technician performing the E-stim knew the exact location of the stimulation contacts and the clinical response evoked, but the researchers who retrospectively viewed the video and recorded the SEEG data in this study were blinded to the location of the stimulation. During E-Stim, the EEG technician are required to monitor the patient’s EEG signals and vital signs to ensure procedural safety. E-Stim procedures must be terminated immediately in the following situations: (1) sustained after-discharges (>5 s); (2) hemodynamic instability; (3) patient-reported adverse symptoms (e.g., pain, chest tightness).

### 2.5 Categories of autonomic cardiac responses

The time interval between two QRS waves (RR interval, RRI) reflects the real-time HR. It was recorded from 5 s before E-stim, and the recording was stopped 5 s after E-stim. For each E-stim, the evoked cardiac responses were divided into three groups based on the RRI alteration (Chouchou et al., 2019): an elevated HR group (E-HR), a declining HR group (D-HR), and a no cardiac response (NS) group. The RRIs within 5 s before E-stim were defined as baseline data, and those within 5 s after stimulation were defined as observation data. A mean of observation data below one standard deviation of the baseline mean was included in the D-HR group, and a mean of observation data one standard deviation above the baseline mean was included in the E-HR group. A mean of observation data within ±1 standard deviation of the baseline mean was included in the NS group (Chouchou et al., 2019).

### 2.6 Statistical methods

Normally distributed data were presented as the mean ± standard deviation, continuous nonparametric data were presented as the median ± interquartile range, and categorical data were presented as frequencies (percentages). Statistical analysis was performed using a one-way analysis of variance (ANOVA) with the least significant difference (Fisher’s least significance difference) for continuous parametric data, Mann–Whitney or Wilcoxon’s signed rank tests for continuous nonparametric data, and Pearson’s chi-square tests for categorical data. Between-group effect sizes and 95% confidence intervals (CIs) were calculated. In the subgroup analysis, extreme RRI values (defined as RRI >1 or RRI <0.4) were excluded and Bonferroni correction was applied to adjust for multiple comparisons across insular subregions. All statistical analyses were conducted using SPSS version 25 (IBM Corporation, Armonk, NY, USA). A two-sided *p* < 0.05 was considered statistically significant.

## 3 Results

### 3.1 Patients

A total of 15 patients were included in this study, 9 patients with ≥1 electrode implanted in the left, 5 in the right, and 1 on both sides. Electrode implantation on the right and left sides of the patient was not significantly different by age or sex. The demographic and clinical characteristics of the patients are presented in Table 1. All patients were right-handed and were not on any medications that affect cardiac contractility or HR. A total of 487 E-stim with 94 electrode contacts were included in the analysis. The mean number of contacts in each person was 6.26. The average stimulation for each electrode contact was 5.18 times. The number of electrode contacts and baseline HRV measurements for all patients are provided in Supplementary material.

**Table 1:** Demographic and clinical features of 15 patients.

Identity	Sex	Age (yr)	dominance	Implanted insula	Seizure onset zone	Medication during E-Stim
P1	M	32	Right-handed	Left	Frontal lobe	Sodium valproate, Clonazepam
P2	M	26	Right-handed	Left	Temporal lobe	Levetiracetam, Oxcarbazepine
P3	F	13	Right-handed	Right	Frontal lobe	Topiramate, Oxcarbazepine, Levetiracetam
P4	F	31	Right-handed	Left	Temporal lobe	Oxcarbazepine
P5	M	12	Right-handed	Left	Parietal lobe	Sodium valproate, Oxcarbazepine
P6	M	16	Right-handed	Left	Frontal lobe	Levetiracetam
P7	F	27	Right-handed	Right	Temporal lobe	Carbamazepine, Sodium valproate
P8	F	32	Right-handed	Right	Temporal lobe	Oxcarbazepine, Sodium valproate
P9	M	18	Right-handed	Both	Parietal lobe	Lacosamide, Perampanel, Levetiracetam
P10	F	38	Right-handed	Left	Cingulated gyrus	Oxcarbazepine
P11	F	25	Right-handed	Left	Frontal lobe	Perampanel, Carbamazepine, Sodium valproate, Levetiracetam
P12	M	15	Right-handed	Right	Temporal lobe	Clonazepam, Sodium valproate
P13	F	29	Right-handed	Left	Temporal lobe	Levetiracetam
P14	M	24	Right-handed	Right	Frontal lobe	Sodium valproate, Oxcarbazepine
P15	M	28	Right-handed	Left	Temporal lobe	Levetiracetam

M, male; F, female. yr, year old.

### 3.2 Cardiac response induced by insula E-stim

First of all, we grouped and localized the E-stim sites based on RRI alteration into E-HR, D-HR, and NS groups. The incidence of HR changes was 40.8% (199) out of 487 E-stims. Ninety-five HR increases after E-stim were included in the E-HR group (57 in the left insula and 38 in the right insula), 104 HR decreases after E-stims were included in the D-HR group (63 in the left insula and 41 in the right insula), and 288 E-stims with no significant HR changes were included in the NS group (125 in the left insula and 163 in the right insula). E-stim of the left insula (120/245) was more likely to elicit HR changes than that of the right side (79/242) (*p* = 0.001, *X*^2^ = 13.443, Cramer’s *V* = 0.166, df = 2), although the trend of these changes did not differ significantly. Of all HR changes elicited by the left insula, 47.5% (57/120) were increases, whereas the proportion was 48.1% (38/79) for the right insula.

### 3.3 Relevance of stimulation parameters to cardiac response

To control for potential confounders affecting cardiac responses, we systematically evaluated the impact of E-Stim parameters (including intensity, inter-pulse interval, and duration) on HR variability. A total of 173 E-stims were administered with low-intensity current, ranging from 1 to 2 mA (30 in E-HR and 26 in D-HR); 164 stimulations were conducted using medium-intensity current between 3 and 4 mA (37 in E-HR and 37 in D-HR); and 150 stimulations were performed with a high-intensity current of 5 to 6 mA (28 in E-HR and 41 in D-HR). We found that low-intensity E-stim tended to increase the HR, whereas high-intensity stimulation had the opposite effect (*p* = 0.034, *X*^2^ = 10.401, Cramer’s *V* = 0.103, df = 4), as shown in Table 2. Additionally, we observed that the mean stimulation intensity was 2.8 ± 1.3 mA, without significant difference between right and left insula (*p* = 0.892). The patient’s age (*p* = 0.477) and gender (*p* = 0.623) were not significantly associated with changes in HR induced by E-Stim. The mean stimulation duration was 4.7 ± 0.6 s and mean inter-stimulation intervals was 22.4 ± 17.2 s. No significant statistical associations were observed between cardiac responses, lateralization, and E-stim parameters (stimulation interval time and duration).

**Table 2:** Number of insular E-Stims by laterality and current intensity.

Lateralization	E-stim intensity	Cardiac response			Total
		E-HR	D-HR	NS	
Left	Low	19	12	48	79
	Middle	24	25	41	90
	High	14	26	36	76
	Total	57	63	125	245
Right	Low	11	14	69	94
	Middle	13	12	49	74
	High	14	15	45	74
	Total	38	41	163	242
Total		95	104	288	487
*p* = 0.001	*p* = 0.034				

E-Stim, electrical stimulation. Effects of laterality (*p* = 0.001, *X*^2^ = 13.44, Cramer’s *V* = 0.166) and current intensity (*p* = 0.034, *X*^2^ = 10.401, Cramer’s *V* = 0.103) on heart rate alteration after insular E-Stim.

### 3.4 Functional mapping of autonomic cardiac responses induced by insula E-stim

**Figure 1:**
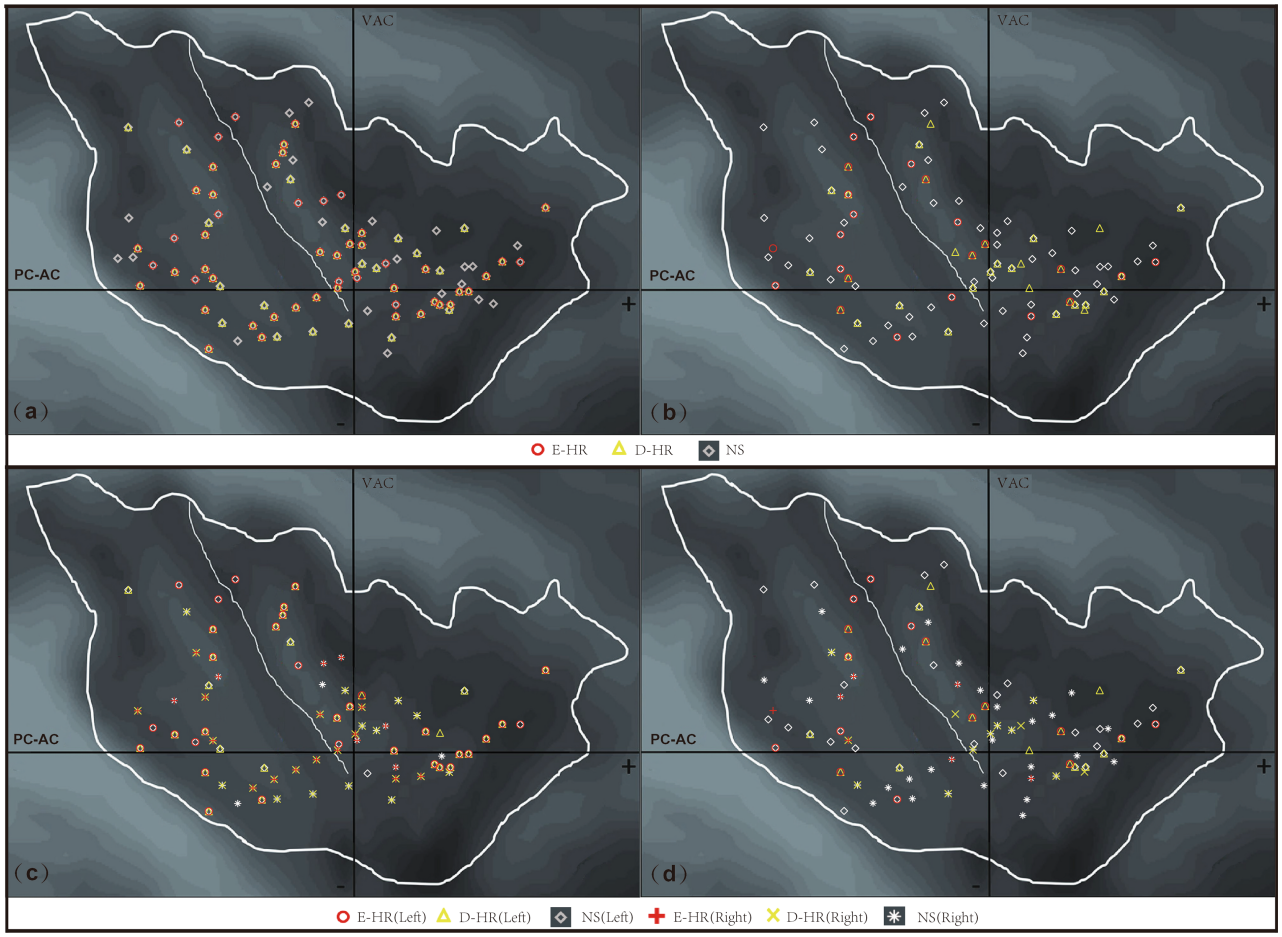
Anatomical distribution of stimulation sites across different HR response patterns following insular E-Stim. **(a)** All stimulation sites across current intensities (D-HR, E-HR, and NS groups combined); **(b)** sites stimulated at moderate intensity (3–4 mA) (D-HR, E-HR, and NS groups combined); **(c)** insular laterality distribution of all stimulation sites across current intensities; **(d)** insular laterality distribution of moderate intensity (3–4 mA) stimulation sites. D-HR, decreased HR group; E-HR, the elevated HR group; NS, no cardiac response group.

**Figure 2:**
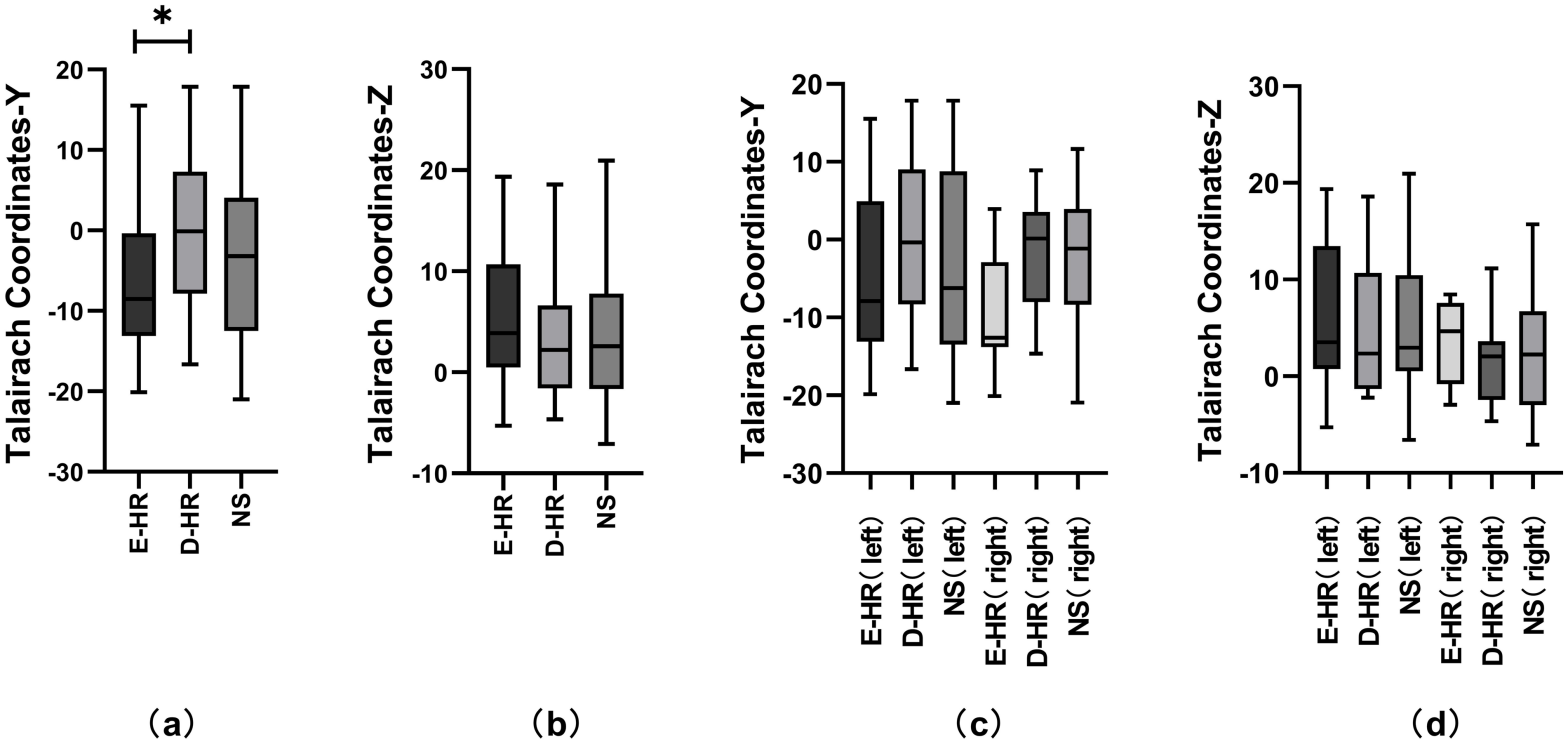
Comparative analysis of Talairach coordinates across different HR change patterns and lateralities. Figures **(a)** and **(b)** represent the comparative analysis of the D-HR, E-HR, and NS groups in the Talairach coordinates Y and Z under the medium-intensity current. Figures **(c)** and **(d)** represent the comparative analysis of the D-HR, E-HR, and NS groups in the left and right insula based on Talairach coordinates Y and Z. Asterisks indicate statistically significant differences (*p* = 0.0419). D-HR, decreased HR group; E-HR, the elevated HR group; NS, no cardiac response group.

Considering the anatomical characteristics of the insula, the sagittal Y and Z coordinates in the Talairach atlas were extracted for analysis in this study (Figure 1a). The localization of the three groups of E-stim was visualized under a medium-intensity current of 3–4 mA (Figures 1b,d) due to the effect of current intensity on HR variability. The laterality data are shown in Figures 1c,d. We found that the distribution of E-stim sites that caused an increase in HR was more posteriorly located in the insula, relative to the sites that decreased HR. There was a statistically significant difference between the E-HR and D-HR groups in the sagittal Y-coordinate (*p* = 0.0419). The Y and Z coordinates in the Talairach atlas of electrode contacts in each group under the medium-intensity current are shown in Figure 2. Statistical analysis based on the Z coordinate axis did not reveal significant differences between the groups.

### 3.5 Middle short gyrus stimulation elicits bradycardic responses

**Figure 3:**
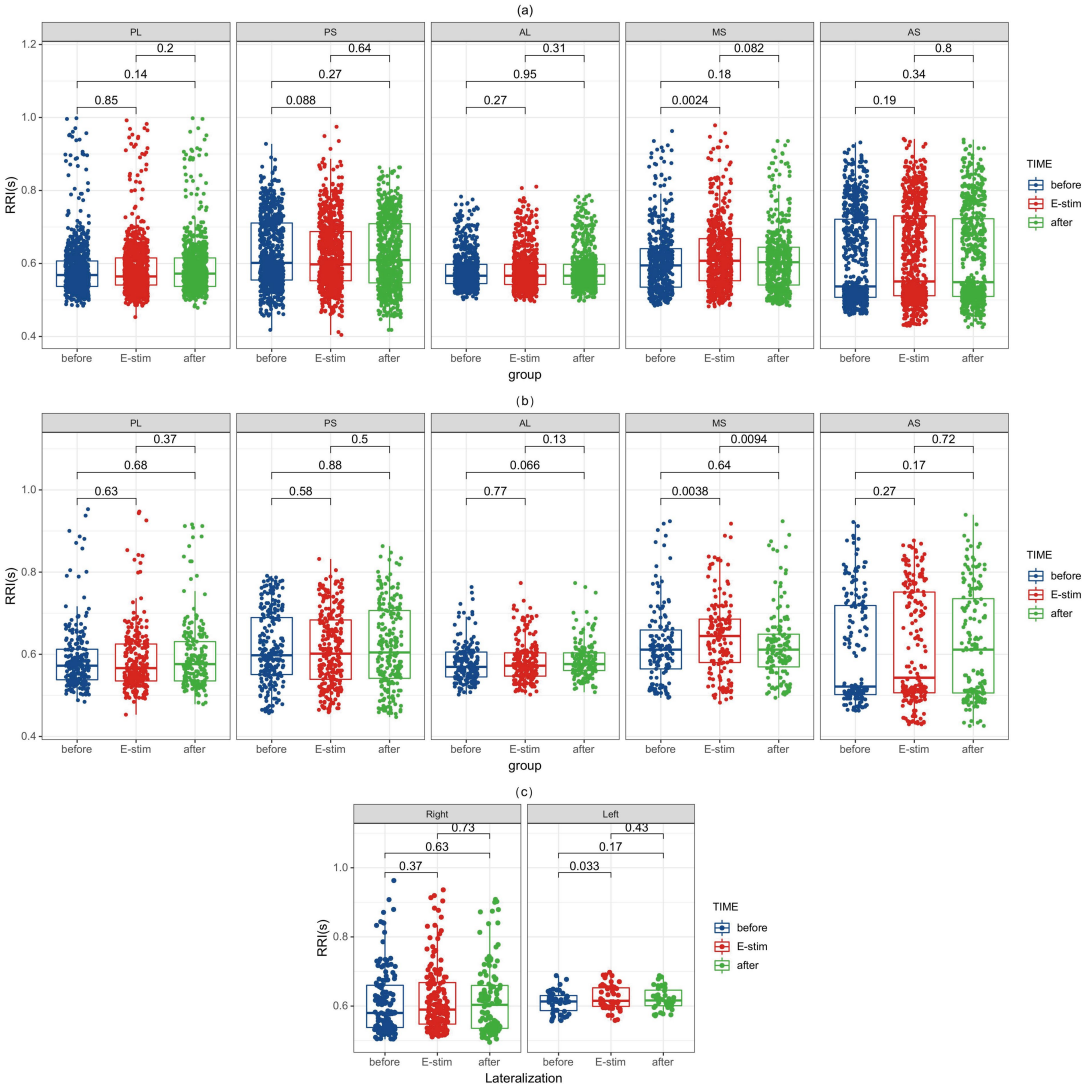
RRI alterations before, during, and after E-Stim of the insular subregion. **(a)** RRI changes evoked by E-Stim at all intensities. **(b)** RRI changes evoked by medium-intensity stimulation. **(c)** RRI changes induced by medium-intensity stimulation of the left versus right middle short gyrus. Notably, significant differences in the middle short gyri survived after Bonferroni correction (corrected *p* = 0.036) in **(a)**. RRI, interval of two QRS wave; E-Stim, electrical stimulation; AS, anterior short gyri; MS, middle short gyri; PS, posterior short gyri; AL, anterior long gyri; PL, posterior long gyri.

Subsequently, based on the observed sagittal-axis differences in stimulation-evoked HR responses across the insula, we conducted targeted analyses of insular subregions (three short insular gyri and two long insular gyri). Two hundred and forty-five stimulations were performed in the left side (73 in the posterior short gyri, 56 in the anterior short gyri, 51 in the anterior long gyri, 45 in the posterior long gyri, and 20 in the middle short gyri) and 242 in the right side (62 in the middle short gyri, 53 in the posterior short gyri, 34 in the anterior short gyri, 49 in the posterior long gyri, and 46 in the anterior long gyri) and phase-specific analysis (pre−/during/post-E-stim) are shown in Figure 3. Our results demonstrated that E-stim of the middle short gyrus elicited significant cardiac deceleration, as evidenced by increased RRI (*p* = 0.0024, *r* = 0.091) during the stimulation; these significant differences remained significant after Bonferroni correction (corrected *p* = 0.036). Under medium-intensity stimulation, E-Stim of the middle short gyrus still elicited a noticeable increase in RRI (*p* = 0.0038, *r* = 0.162), which rapidly returned to baseline after cessation. However, this effect did not survive Bonferroni correction (corrected *p* = 0.057). Further stratification by hemisphere revealed that stimulation of the left middle short gyrus induced a statistically significant increase in HR (*p* = 0.033, *r* = 0.142), an effect not observed on the right side. There was no statistically significant difference in RRI alteration in other insula subdivisions.

## 4 Discussion

To the best of our knowledge, this is the first study to summarize the role of the middle short gyrus in insula in cardiac rhythm regulation in a large-scale SEEG-based study with 487 E-stims. In summary, 40.8% (199/487) of insula stimulations evoked a cardiac response, of which 19.5% (95/487) increased the HR and 21.3% (104/487) decreased the HR. The laterality of insular stimulation and current intensity were independently associated with both the incidence and directionality of evoked HR alterations. Compared to the distribution of the insula electrode contacts in the D-HR group, those in the E-HR group were predominantly distributed posterior to the insula under the same current intensity. Most notably, we pinpointed a specific functional subregion: E-Stim of the middle short gyrus robustly and specifically elicited significant cardiac deceleration.

The interaction of the vagus and sympathetic nerves is key to HR regulation and underlies the rhythmic contraction of the heart. While the classic literature on neurocardiology focused on the role of subcortical regions of the central ANS in the regulation of HR, more recent studies have suggested that the cerebral cortex can also regulate the ANS by acting on subcortical structures, including the insula (Benarroch, 2020), cingulate gyrus (Kim et al., 2019), hypothalamus (Henderson and Macefield, 2021), zona incerta (Messina and Overton, 2007), and frontal lobe (Harnod et al., 2009), to affect the HR. A few studies have reported the characteristics of HR changes after E-stim of the insula cortex. Oppenheimer and Cechetto (1990) found earlier in rats that E-stim of posterior insula cortical areas affected HR. In addition, a link between insula neural activity and HR has also been observed by non-invasive techniques (Legon et al., 2023, 2024). Owing to advancements in SEEG technology, direct observation of HR changes induced by E-stim in the human insula has become feasible in recent years. A retrospective study analyzing 100 E-stims performed on patients with epilepsy found that 90% of stimulation-induced clinical sensations, and 47% induced changes in HR (Chouchou et al., 2019). However, these findings cannot exclude potential confounding effects of stimulation-evoked paresthesia and current intensity on cardiac responses, particularly given our demonstration of significant current intensity-dependent HR modulation. The distribution and prevalence of E-Stim sites associated with HR variability are consistent with the results of the present study. Sanchez-Larsen et al. (2021) eliminated sensory confounders and reported predominant HR deceleration following insular stimulation (*n* = 100); however, their study neither identified potential tachycardia-inducing stimulation sites nor accounted for the significant interindividual variability inherent due to real-time HR monitoring analysis. The 40.8% incidence of HR alteration and the anatomical distribution of the relevant insula stimulation sites in our study of 487 insula E-stims, after excluding stimulation sites that had induced clinical symptoms and unifying the intensity of E-stim, remained broadly consistent with previous findings.

Current evidence remains controversial regarding the functional lateralization and subdivision-specific modulation of HR by the insular cortex. Leona et al. demonstrated a significant positive correlation between resting HR and the left insular cortex volume, contrasting with a significant negative correlation for the right insular cortex volume (Danilin et al., 2022). Nevertheless, animal experiments, revealed symmetrical cardiovascular responses (including blood pressure changes, HR changes, or renal sympathetic nerve activity) to chemical stimulation of bilateral insular cortices (Marins et al., 2016). In our study, the direction of HR response induced by insular E-stim is not significantly associated with insular lateralization, although the left insula appeared more prone to elicit cardiac reactions, which aligns with previous animal experimental findings. Notably, low-current-intensity stimulation tended to increase HR, while high-intensity stimulation produced the opposite effect. We cannot exclude the possibility that the HR elevation during low-current E-stim reflects an anxiety response to the initial phase in the experimental procedure rather than a direct current effect (Martha et al., 2021); conversely, high-current stimulation elicited more robust cardiovascular responses that were likely unaffected by external confounders and thus more accurately reflected the true cortical stimulation effects. These intensity-dependent observations corroborate Sanchez-Larsen et al.’s (2021) findings demonstrating more substantial HR alterations associated with higher-intensity insular stimulation. Additionally, the higher activation thresholds of autonomic or nociceptive fibers may lead to unintended stimulation of non-target nerve fibers (Umair et al., 2022), potentially introducing confounding effects on HR alteration through current spread to adjacent brain regions. Concurrently, antiepileptic drugs (AEDs) are known to modulate autonomic function in epilepsy patients. Gradual AEDs withdrawal has been associated with enhanced parasympathetic and sympathetic tone, accompanied by increased HRV (Lossius et al., 2007). Carbamazepine has been shown to induce significant systolic blood pressure changes during Valsalva maneuvers, indicating reduced sympathetic reactivity (Francesca et al., 2020). Lamotrigine has been documented to prolong QT intervals (Kevan and Djans, 2007). However, under experimental conditions comparable to the present study—whether comparing different monotherapy regimens (Francesca et al., 2020) or contrasting monotherapy versus polytherapy approaches (Omnia Fathy et al., 2015)—the observed alterations in HRV parameters remain relatively limited.

In our study, the posterior insula evoked a greater increase in HR, while the anterior part evoked a greater decrease. The insular cortex can be anatomically and functionally subdivided into three distinct subregions—the posterior granular, middle dysgranular, and anterior agranular areas—based on cytoarchitectonic organization, connectivity patterns, and functional specialization (Benarroch, 2019). In the present study, we demonstrate a clear functional dichotomy within this structure: E-Stim of the anterior agranular insular cortex elicits pronounced bradycardia, likely mediated through parasympathetic pathways (Chouchou et al., 2019), while activation of the posterior granular insular region produces significant tachycardia, presumably via sympathetic modulation. These findings align with previous neuroanatomical evidence (van der Kooy et al., 1984) that identified potential direct projections from the granular insular zone to the dorsal motor nucleus of the vagus nerve using fluorescent retrograde tracing techniques, further supporting the concept of topographically organized autonomic control within distinct insular subregions. Further analysis revealed that HR showed an immediate and significant decrease following stimulation onset in the middle short insular gyri, with a recovery trend observed within 5 s post-stimulation. In contrast, no significant HR variations were detected following stimulation of other insular subregions. These findings provide the first evidence that the middle short insular gyri may serve as the key functional locus within the anterior insula responsible for mediating HR deceleration. The study by Shinpei et al. (2016) demonstrates that the middle short gyrus serves as an upstream hub within a functional neural circuit involving the anterior short gyrus (ASG), which collectively orchestrates viscero-autonomic reflexes. Neurofunctional mapping studies have localized the middle short gyrus as the predominant cortical region responsible for insula-mediated functions, including pain perception (Afif et al., 2008) and speech production (Afif et al., 2009).

Neurocardiac damage is strongly associated with sudden unexpected death and increased perioperative risks in epilepsy surgery (Oveisgharan et al., 2022; Laoprasert et al., 2017). Notably, paroxysmal tachycardia or bradycardia frequently occurs in cases of insular epilepsy (Sami et al., 2017). Investigating the functional mapping between insular subregions and HR alteration may enhance the precision of epileptogenic zone localization while preserving critical autonomic neural circuits, thereby supporting cardiovascular homeostasis during the perioperative period. Importantly, structural or functional insular impairment—whether due to epileptic foci (Lacuey et al., 2016), stroke (Romano et al., 2019), or surgical resection (de Morree et al., 2016)—elevates mortality risk. The incidence of SUDEP is estimated to range from 0.7 to 1.3 per 1,000 patient-years, escalating to 3.5–4.1 per 1,000 patient-years in those with refractory epilepsy (Buchanan et al., 2023; Walczak et al., 2001). Several clinical studies have demonstrated a correlation between SUDEP, HR variability, and the insula (Kassinopoulos et al., 2023; Scorza et al., 2022). In a 3-year longitudinal study of 320 patients, two cases of SUDEP were observed, both involving individuals with prior insular injury who exhibited progressive HRV deterioration preceding death (Lacuey et al., 2016). To date, the pathophysiology of SUDEP remains incompletely understood, though it often involves bradyarrhythmia progressing to asystole and fatal cardiac arrest (Devinsky et al., 2016; Li et al., 2017). In our study, we found that the middle short insular gyri may be a key node for triggering bradycardia in the HR regulation pathway. Future research should conduct prospective, causally designed clinical trials based on closed-loop stimulation or telemetry-integrated monitoring to specifically assess the therapeutic potential of the middle short insular gyri in preventing SUDEP and improving the efficacy of epilepsy surgery.

The present study has several limitations. First, it is a retrospective, observational study that could not standardize all individual-specific factors, including AEDs, stimulation parameters, and electrode placement. Consequently, we could not fully control for confounding factors arising from patient heterogeneity, individual neuroanatomical variations, and differences in electrode coverage. Second, potential confounding factors from procedural stress on HRV could not be completely eliminated. Third, the spatial resolution of E-Stim is limited by current spread to adjacent gyri or subcortical regions, potentially causing nonspecific activation. Fourth, applying mirror processing could potentially obscure these biologically meaningful hemispheric differences. Furthermore, we did not monitor cardiac function, blood pressure, and respiratory function, which precludes analysis of these additional autonomic parameters and may reduce ECG signal quality. Compared with traditional leads, deltoid leads are inferior in terms of electrical signal quality and artifacts. In the future, the use of complete electrocardiogram detection equipment synchronized with EEG signals will improve data quality and reliability, and the short E-stim durations and intervals cannot reveal long-term autonomic effects in this study. Lastly, there were multiple E-Stims originating from the same site or the same patient, which may have introduced clustering-related bias into the analysis results. Future studies could design prospective experiments with stratified analysis to eliminate any existing bias as much as possible.

## 5 Conclusion

Approximately 40.8% of insular E-stim induced a cardiac response (19.5% increased the HR and 21.3% decreased the HR). Our findings suggest that insula subdivisions and lateralization may play an important role in the regulation of HR. Stimulation sites inducing HR elevation were anatomically localized to posterior insular regions relative to bradycardic response. Among these, we found a correlation between the middle short gyrus and HR reduction. This study is the first to observe the role of the middle short gyrus in cardiac rhythm regulation and may provide a new direction for pre-surgical assessment of epilepsy and the prevention and treatment of SUDEP.

## Data Availability

The raw data supporting the conclusions of this article will be made available by the authors, without undue reservation.
