# Supplementary material for: Topography of the insular cortex in heart rate control: high-precision mapping reveals critical role of the middle short gyrus
Source: Front Neurosci. 2025 Sep 17;19:1665378. doi: 10.3389/fnins.2025.1665378 (PMC12484038; doi:10.3389/fnins.2025.1665378)
Supplement: Supplementary file 1 [file Table_1.DOCX]

Supplementary 1 Electrode contacts number, location and HRV baseline of patients.

| Identity | Electrode contacts number | Location | | | | | RMSSD  (ms) | SDNN  (ms) |
| --- | --- | --- | --- | --- | --- | --- | --- | --- |
|  |  | AS | MS | PS | AL | PL |  |  |
| P1 | 2 | - | - | 2 | - | - | 97.3 | 75 |
| P2 | 5 | - | - | 3 | - | 2 | 8 | 29.5 |
| P3 | 12 | - | 2 | 1 | 4 | 5 | 21.9 | 38.7 |
| P4 | 19 | 5 | 1 | 4 | 5 | 4 | 35.4 | 30.4 |
| P5 | 3 | - | 3 | - | - | - | 55.4 | 75.2 |
| P6 | 13 | 3 | 5 | 5 | - | - | 55.4 | 75.2 |
| P7 | 3 | 3 | - | - | - | - | 31.8 | 41.7 |
| P8 | 4 | 4 | - | - | - | - | 20.6 | 58.0 |
| P9 | 4 | - | 4 | - | - | - | 33.3 | 40.2 |
| P10 | 8 | - | - | - | 4 | 4 | 51.1 | 68.3 |
| P11 | 2 | - | - | - | - | 2 | 35.1 | 25.7 |
| P12 | 9 | 3 | - | 4 | 2 | - | 17.9 | 12.9 |
| P13 | 3 | - | - | 3 | - | - | 26.3 | 20.2 |
| P14 | 5 | 2 | - | 3 | - | - | 89.7 | 89.8 |
| P15 | 2 | 2 | - | - | - | - | 88.7 | 88.2 |

HRV, heart rate variability; RMSSD, Root Mean Square of Successive Differences; SDNN, Standard Deviation of Normal-to-Normal intervals; AS, anterior short gyri; MS, middle short gyri; PS, posterior short gyri; AL, anterior long gyri; PL, posterior long gyri.
